# Vitamin C Supplementation in Hospitalized Patients With Community-Acquired Pneumonia: Protocol for a Randomized Controlled Trial

**DOI:** 10.2196/91037

**Published:** 2026-04-29

**Authors:** Yogesh Sharma, Arduino A Mangoni, Richard Woodman, Huah Shin Ng, Manisha Arthur, Subodha Sumanadasa, Shailesh Bihari, Campbell Thompson

**Affiliations:** 1 College of Medicine & Public Health Flinders University Adelaide, SA Australia; 2 Department of Acute and General Medicine Flinders Medical Centre Adelaide, SA Australia; 3 Intensive and Critical Care Unit Flinders Medical Centre Adelaide, SA Australia; 4 Discipline of Medicine The University of Adelaide Adelaide, South Australia Australia

**Keywords:** randomized controlled trial, community-acquired pneumonia, vitamin C supplementation, clinical stabilization, adjunctive therapy

## Abstract

**Background:**

Community-acquired pneumonia (CAP) remains a leading cause of hospitalization, morbidity, and mortality worldwide, particularly among older adults with multimorbidity and frailty. Despite advances in antimicrobial therapy, clinical outcomes have improved little, highlighting the need for safe, inexpensive adjunctive treatments. Vitamin C plays a critical role in immune function, redox homeostasis, and endothelial integrity, all disrupted during acute infection. Hypovitaminosis C is common in hospitalized patients with CAP and has been associated with increased disease severity, longer length of stay (LOS), and worse outcomes. However, prior randomized trials of vitamin C have produced inconsistent results, often focusing on critically ill patients with sepsis, using short treatment durations, and discontinuing therapy abruptly.

**Objective:**

The Vitamin C in Community-Acquired Pneumonia (VitCAP) trial aims to evaluate whether high-dose oral vitamin C administered over an extended period improves clinical recovery and patient-centered outcomes in adults hospitalized with CAP.

**Methods:**

VitCAP is a single-center, double-blind, placebo-controlled, parallel-group randomized clinical trial conducted at a tertiary hospital in Australia. Adults aged 18 years and older hospitalized with CAP will be randomized within 48 hours of admission in a 1:1 ratio to receive either oral sodium ascorbate (1 g 3 times daily for 7 days, followed by 500 mg twice daily for 30 days) or a matching placebo in addition to standard care. Randomization will be computer generated with allocation concealment via a centralized pharmacy service, and all participants, clinicians, investigators, and outcome assessors will remain blinded. The primary outcome is time to clinical stabilization, defined using standard physiological criteria. Secondary outcomes include early clinical response, symptom burden at 30 days, intensive care unit admission, need for ventilatory or vasopressor support, LOS, all-cause mortality at 30 days and 6 months, hospital readmission, health-related quality of life, and changes in inflammatory biomarkers (C-reactive protein and procalcitonin). Analyses will follow the intention-to-treat principle. The primary outcome will be analyzed using Cox proportional hazard regression adjusted for prespecified covariates, with sensitivity analyses including restricted mean survival time.

**Results:**

The VitCAP trial received ethics approval from the Southern Adelaide Local Health Network Human Research Ethics Committee in 2025 and funding in September 2025. Recruitment is expected to commence in 2026 and continue for 18 to 24 months. A total of 124 participants will be enrolled to provide 80% power to detect a clinically meaningful difference in time to clinical stabilization while allowing for attrition. Data analysis will follow completion of follow-up, with primary results anticipated in 2028.

**Conclusions:**

The VitCAP trial is designed to address important evidence gaps by evaluating sustained oral vitamin C supplementation in hospitalized patients with CAP using clinically meaningful patient-centered outcomes. If effective, vitamin C could represent a safe, low-cost, and scalable adjunct to standard CAP management.

**Trial Registration:**

Australian New Zealand Clinical Trials Registry (ANZCTR) ACTRN12625001361493; https://anzctr.org.au/Trial/Registration/TrialReview.aspx?ACTRN=12625001361493

**International Registered Report Identifier (IRRID):**

PRR1-10.2196/91037

## Introduction

### Background

Community-acquired pneumonia (CAP) is an acute infection of the pulmonary parenchyma that occurs outside the hospital setting and remains a major cause of hospitalization, morbidity, and mortality worldwide. Lower respiratory tract infections accounted for approximately 2.6 million deaths worldwide in 2019, ranking among the leading causes of death [1]. Population-based CAP studies from high-income countries report hospitalization rates of approximately 25 per 10,000 adults annually, with 20% to 25% of patients requiring intensive care unit (ICU) admission [2]. Mortality remains substantial, particularly among older adults and those with multimorbidity and frailty [3].

Recent Australian data highlight the persistent burden of CAP. In a large multicenter cohort of patients hospitalized with non–COVID-19 CAP, in-hospital and 30-day mortality rates were 7.8% and 16.9%, respectively, rising to 17.6% among those requiring ICU admission [4]. Despite advances in antimicrobial therapy and supportive care, outcomes have remained largely unchanged, underscoring the need for safe and effective adjunctive therapies to improve recovery and reduce complications.

### Rationale for Vitamin C Supplementation

Inflammation and oxidative stress are central to the pathophysiology of CAP and its complications, including respiratory failure and sepsis-related organ dysfunction, through the excessive production of reactive oxygen species and proinflammatory cytokines, with consequent tissue injury [5-7]. Vitamin C (ascorbate) is a key antioxidant with immunomodulatory properties; however, humans cannot synthesize it endogenously and rely entirely on dietary intake [8].

Hypovitaminosis C is common in acute illness due to increased metabolic demand, reduced intake, and enhanced losses and has been associated with worse outcomes in sepsis and pneumonia [9-11]. Among hospitalized patients with CAP, our research [12] suggests that 42.6% are vitamin C deficient and these patients had 23% longer hospital length of stay (LOS; incidence rate ratio=1.23, 95% CI 1.06-1.42; *P*=.005) and 3-fold longer ICU LOS (incidence rate ratio=3.07, 95% CI 2.18-4.30; *P*<.001) after adjustment for age, comorbidities, frailty status, and pneumonia severity when compared to patients who were not deficient. There was a trend toward increased in-hospital mortality (adjusted odds ratio 4.23, 95% CI 0.58-30.48) and 30-day mortality (adjusted odds ratio 3.82, 95% CI 0.71-20.50) among patients who had vitamin C deficiency. Randomized trials evaluating vitamin C in the treatment of infections have yielded inconsistent results. While some studies suggest improvements in respiratory parameters, larger trials in patients with sepsis who are critically ill have not demonstrated a clear mortality benefit, potentially due to differences in patient populations, formulation, dosing strategy, and abrupt discontinuation of therapy [13-15]. Furthermore, evidence specific to hospitalized patients with CAP outside the ICU remains limited.

### Study Justification

Previous randomized controlled trials (RCTs) evaluating vitamin C in CAP have yielded inconsistent findings, with most research focused on patients with sepsis who are critically ill rather than hospitalized CAP populations [14,16,17]. A 2021 systematic review including 5 RCTs of oral and parenteral vitamin C reported modest reductions in time to improvement in oxygen saturation and respiratory rate, suggesting potential clinical benefit [13]. In contrast, the Lessening Organ Dysfunction With Vitamin C (LOVIT) trial, which evaluated high-dose intravenous ascorbic acid in patients with sepsis who were critically ill (one-third with respiratory infection), found no significant mortality benefit, although treatment was abruptly discontinued and ascorbic acid was used rather than sodium ascorbate [14]. Subsequent meta-analyses incorporating LOVIT trial data have reported low-certainty evidence, with point estimates favoring reductions in in-hospital (risk ratio [RR]=0.88, 95% CI 0.73-1.06) and 30-day (RR=0.83, 95% 0.71-0.98) mortality. Our recent CAP-specific systematic review demonstrated a nonsignificant trend toward reduced mortality (RR=0.51, 95% CI 0.24-1.09; *P* for overall effect =.05; *I*^2^*=*0%; *P* for heterogeneity=.65) [18,19]. The absence of benefit in LOVIT and similar trials may relate to rebound hypovitaminosis following abrupt cessation, increased metabolic consumption during sepsis, or formulation-related effects, as also observed in the Vitamin C Infusion for Treatment in Sepsis Induced Acute Lung Injury (CITRIS-ALI) study [20]. Overall, existing studies [14,17] are predominantly ICU based, frequently involve abrupt treatment discontinuation, and rarely assess patient-centered outcomes or symptom recovery. Therefore, expert consensus [16,20,21] highlights the need for rigorously designed trials evaluating sustained vitamin C replacement using sodium ascorbate in hospitalized patients with CAP, providing a strong justification for this study.

Thus, there is a clear evidence gap regarding the effectiveness of vitamin C supplementation as an adjunct therapy in hospitalized patients with CAP, particularly using oral sodium ascorbate and assessing patient-centered outcomes. The Vitamin C in Community-Acquired Pneumonia (VitCAP) trial is designed to address this gap by evaluating high-dose oral vitamin C administered over an extended period; avoiding abrupt cessation; and focusing on clinically meaningful end points, including time to clinical stabilization, symptom recovery, and health-related quality of life (HRQOL). This study aims to generate high-quality evidence to inform future clinical practice and guideline development.

### Study Aim and Hypotheses

The primary aim of the VitCAP trial is to determine whether high-dose oral vitamin C (sodium ascorbate) administered during hospitalization and continued for 30 days after discharge improves clinical recovery in adults hospitalized with CAP when compared with placebo alongside standard care. The primary hypothesis is that vitamin C supplementation will reduce the time to clinical stabilization compared with placebo.

Secondary hypotheses are that vitamin C supplementation will (1) improve early clinical response; (2) reduce hospital LOS; (3) decrease the need for intensive care, ventilatory support, or vasopressors; (4) reduce mortality and hospital readmission; (5) improve patient-reported symptom burden and HRQOL; and (6) reduce systemic inflammatory biomarkers, including C-reactive protein and procalcitonin.

## Methods

### Study Design

**Figure 1:**
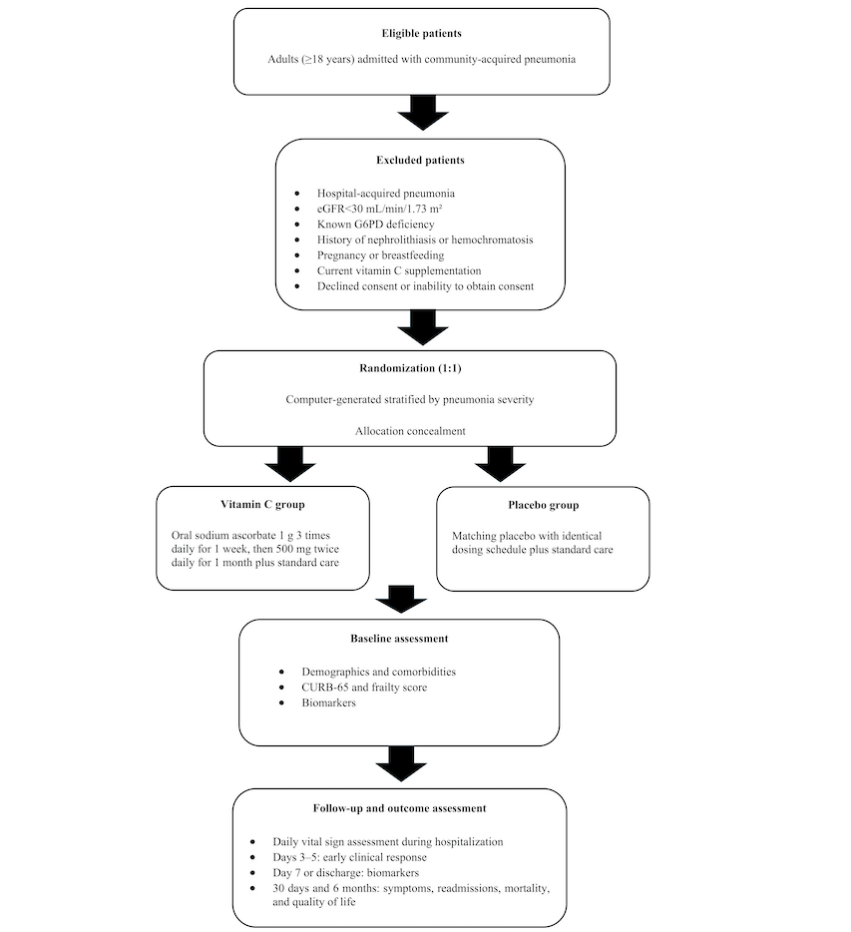
Flow diagram for the Vitamin C in Community-Acquired Pneumonia trial. CURB-65: confusion, urea, respiratory rate, blood pressure, and age ≥65 years; eGFR: estimated glomerular filtration rate; G6PD: glucose-6-phosphate dehydrogenase.

This study is a single-center, double-blind, placebo-controlled, parallel-group randomized clinical trial designed in accordance with the SPIRIT (Standard Protocol Items: Recommendations for Interventional Trials) 2013 statement. Participants will be randomly allocated in a 1:1 ratio to receive either high-dose oral vitamin C (as sodium ascorbate) or a matching placebo in addition to standard care for CAP (Figure 1).

### Study Setting

The trial will be conducted at a tertiary hospital in Australia providing acute general medical and respiratory services. Recruitment will be conducted among patients admitted under general medicine, respiratory, or intensive care teams with a primary diagnosis of CAP.

### Eligibility Criteria

The eligibility criteria have been provided in Textbox 1.

**Eligibility criteria.**

**Inclusion criteria**

- Age of 18 years or older
- Hospital admission with community-acquired pneumonia (clinical symptoms of cough, sputum production, dyspnea, and fever, along with imaging evidence of an infiltrate)
- Randomization within 48 hours of hospital admission
- Ability to provide informed consent or availability of a legally authorized representative

**Exclusion criteria**

- Hospital-acquired or ventilator-associated pneumonia
- Severe renal impairment (estimated glomerular filtration rate <30 mL per minute per 1.73 m^2^)
- Known glucose-6-phosphate dehydrogenase deficiency
- History of nephrolithiasis or hemochromatosis
- Pregnancy or breastfeeding
- Current use of vitamin C supplements

### Interventions

Participants allocated to the intervention arm will receive *oral sodium ascorbate* (*1 g 3 times daily for 7 days,* followed by *500 mg twice daily for 30 days*). The control group will receive a matching placebo administered using the same dosing schedule. The placebo consists of capsules containing Starch 1500 (partially pregelatinized maize starch; Colorcon, Inc) as an inert excipient. The placebo material will be encapsulated in white opaque size 0 Coni-Snap hard gelatin capsules (Lonza). Active and placebo capsules will be matched in size, color, and appearance to maintain blinding. Adherence will be assessed through medication administration records during hospitalization and participant self-report after discharge.

All participants will receive standard CAP management in accordance with local clinical guidelines. Standard management for hospitalized patients with CAP at the study center follows national and local clinical guidelines. Initial management typically includes early administration of empirical antibiotics according to pneumonia severity and risk factors and supportive care, including oxygen therapy when required, intravenous fluids, venous thromboembolism prophylaxis, and monitoring of vital signs and laboratory markers of infection. Patients with severe disease may receive care in the intensive care settings and may require respiratory or hemodynamic support.

The vitamin C dosing regimen for this trial was selected to rapidly correct hypovitaminosis C during the acute illness phase (1 g 3 times daily for 7 days) and maintain adequate plasma concentrations during recovery (500 mg twice daily for 30 days). Oral sodium ascorbate was chosen for its high bioavailability, tolerability, and practicality for prolonged administration in both hospital and postdischarge settings. This approach avoids abrupt cessation and potential rebound deficiency observed in previous trials such as LOVIT [14] and CITRIS-ALI [20].

### Outcomes

The primary outcome is *time to clinical stabilization*, defined as the time from randomization to meeting all the criteria.

The primary and secondary outcomes have been provided in Textbox 2.

**Primary and secondary outcomes.**

**Primary outcome**

- Temperature of 37.8 °C or less
- Heart rate of 100 beats per minute or less
- Respiratory rate of 24 breaths per minute or less
- Systolic blood pressure of 90 mm Hg or higher
- Oxygen saturation of 90% or higher or arterial partial pressure of oxygen of 60 mm Hg or higher on room air

**Secondary outcomes**

- Early clinical response [22], defined as improvement in at least 2 of 4 specific symptoms (chest pain, cough, sputum production, and dyspnea) without any symptom worsening 3 to 5 days after randomization
- Improvement in community-acquired pneumonia–related symptoms at 30 days from the day of randomization, measured using the 18-item community-acquired pneumonia symptom questionnaire [23] (range 0-90)
- Intensive care unit admission
- Requirement for noninvasive or invasive mechanical ventilation
- Vasopressor use
- Length of hospital stay
- All-cause mortality at 30 days and 6 months
- 30-day hospital readmission
- Health-related quality of life at 1 and 6 months using the EQ-5D-5L [24]
- Inflammatory biomarkers (C-reactive protein and procalcitonin) measured at baseline and day 7 or discharge

### Sample Size Calculation

The sample size was based on a previous RCT [16], which reported that the mean time to clinical stabilization (used as a proxy for hospital discharge) in patients with CAP was 3.6 (SD 1.9) days in patients who were in the vitamin C group compared to 4.7 (SD 2.0) days in the placebo group. Using this effect size (1.1 days), a pooled SD of 1.95, a power of 80%, and a 2-sided α of .05, the required sample size is 102 participants (51 per group). To account for an anticipated 20% attrition rate due to loss to follow-up or mortality, we plan to recruit 124 participants (62 per group).

### Randomization and Allocation Concealment

Randomization will be performed using a computer-generated sequence with variable block sizes and stratification by pneumonia severity. Allocation concealment will be ensured via a centralized pharmacy service responsible for dispensing sequentially numbered, identical study medications.

### Blinding

Participants, treating clinicians, investigators, outcome assessors, and statisticians will remain blinded to treatment allocation. Study medications will be indistinguishable in appearance, taste, and packaging. Emergency unblinding will be permissible only when knowledge of allocation is essential for clinical management.

### Data Collection and Follow-Up

Clinical data will be collected daily during hospitalization. Follow-up assessments will be conducted at 30 days and 6 months after randomization via telephone interview. Data will be recorded using secure electronic case report forms. The schedule of enrollment, interventions, and outcome assessments is summarized in Table 1.

**Table 1:** Schedule of enrollment, interventions, and assessments for the Vitamin C in Community-Acquired Pneumonia trial.

	Screening and baseline	Day 0—randomization	Days 1-7—hospital phase	Day 30—follow-up	Month 6—follow-up
Eligibility assessment	✓				
Informed consent	✓				
Randomization		✓			
Vitamin C or placebo administration		✓	✓	✓	
Clinical observations (vital signs)	✓	✓	✓^a^		
CAP^b^ symptom assessment	✓		✓	✓	
Early clinical response assessment			✓^c^		
Inflammatory biomarkers (CRP^d^ and PCT^e^)	✓		✓^f^		
Hospital outcomes (ICU^g^ admission, ventilation, and vasopressor use)			✓		
Length of stay			✓		
Adverse event monitoring		✓	✓	✓	✓
Hospital readmission				✓	
Health-related quality of life (EQ-5D-5L)				✓	✓
Mortality assessment			✓	✓	✓

^a^Daily during hospitalization (days 1-7).

^b^CAP: community-acquired pneumonia.

^c^Assessed on days 3-5.

^d^CRP: C-reactive protein.

^e^PCT: procalcitonin.

^f^Measured on day 7 or at discharge (whichever occurs first).

^g^ICU: intensive care unit.

### Statistical Methods

The primary analysis for this trial will be based on the intention-to-treat principle and will include all randomized patients, with treatment effects based on the assigned treatment regardless of the treatment they received and whether they completed the trial.

#### Primary Outcome Analysis

The primary outcome, time to clinical stabilization, will be analyzed using a Cox proportional hazard regression model with treatment group as the primary exposure. Results will be reported as hazard ratios with 95% CIs accounting for censoring. The proportional hazard assumption will be assessed using Schoenfeld residuals and log-log survival plots.

The primary Cox model will be adjusted for the following prespecified baseline covariates selected a priori based on clinical relevance: age, sex, baseline pneumonia severity (confusion, uremia, respiratory rate, blood pressure, and age ≥65 years [CURB-65] score), baseline C-reactive protein concentration, and key comorbidities (eg, chronic cardiovascular, respiratory, or metabolic conditions). These covariates were selected to account for potential confounding effects of multimorbidity on clinical recovery. Randomization is stratified by pneumonia severity to ensure balance between groups across this critical prognostic variable.

As a sensitivity analysis, time to clinical stabilization will also be assessed using restricted mean survival time over a clinically relevant time horizon, providing an absolute measure of treatment effect that does not rely on the proportional hazard assumption.

An additional sensitivity analysis will be performed using an independent-sample *t* test (2-sided) or linear regression depending on the distribution of the outcome. The linear regression model will allow for adjustment for the same prespecified covariates, and results will be reported as mean differences with 95% CIs.

#### Secondary Outcome Analysis

Hospital LOS will be analyzed similarly using *t* tests or linear regression, with Cox regression as a secondary analysis. Although LOS data are likely to be nonnormally distributed, the *t* test and linear regression models are considered robust due to the sample size (n>30 per group), which satisfies the conditions of the central limit theorem.

HRQOL at 1 and 6 months will be assessed using linear mixed-effects models to account for repeated measures, and binary outcomes (eg, ICU admission and in-hospital mortality) will be compared using logistic regression. Biomarkers will be analyzed as change from baseline with adjustment for time from randomization to follow-up sampling. Linear regression and mixed-effects models incorporating time as a covariate will be used, with sensitivity analyses restricted to samples collected within a predefined time window.

Baseline differences between groups in clinical and demographic variables will be assessed using *t* tests, Wilcoxon rank-sum tests, or chi-square or Fisher exact tests as appropriate. Missing data will be addressed using multiple imputation, and additional sensitivity analyses will be performed in subgroups, such as patients with moderate to severe pneumonia (CURB-65 score≥2).

Statistical significance for all outcomes will be determined using a 2-sided type I error rate of α=.05. All analyses will be performed using Stata (version 19.0; StataCorp).

#### Subgroup Analyses

Prespecified subgroup analyses will examine the effects of vitamin C in clinically important subgroups, including older participants (aged <65 years vs ≥65 years), participants with frailty (Clinical Frailty Scale score of 1-5 vs ≥5), participants with moderate to severe pneumonia (CURB-65 score of <2 vs ≥2), and participants with higher multimorbidity burden (Charlson Comorbidity Index of <3 vs ≥3). Separate regression models will be performed for each subgroup.

### Safety Monitoring

#### Adverse Events

An adverse event (AE) is any untoward medical occurrence, including symptoms, signs, illnesses, or abnormal laboratory findings, that develops or worsens during the trial irrespective of causality. All AEs will be recorded from randomization until completion of follow-up.

#### Serious AEs

A serious AE (SAE) is any AE that results in death, is life-threatening, requires or prolongs hospitalization, results in persistent or significant disability or incapacity, causes congenital anomalies, or constitutes an important medical event in the judgment of the investigators.

#### Unanticipated Problems

Unanticipated problems include any unexpected incident, experience, or outcome related or possibly related to trial participation that suggests increased risk of harm to participants or others.

#### Serious and Unexpected Adverse Drug Reactions

Serious and unexpected adverse drug reactions are noxious and unintended responses to the study drug that meet SAE criteria and are inconsistent with existing product information.

#### Reporting Procedures

All SAEs, serious and unexpected adverse drug reactions, and unanticipated problems will be reported to the chief investigator within 24 hours of awareness. The trial center will report events to relevant regulatory authorities within 7 days for fatal or life-threatening events and within 15 days for other serious events. Nonserious adverse reactions will be recorded and monitored.

#### Data Safety Monitoring Board

An independent data safety monitoring board comprising clinicians and a biostatistician will oversee participant safety and trial conduct. The data safety monitoring board will periodically review aggregated safety data and may recommend trial continuation, modification, or termination based on safety concerns.

#### Discontinuation of Study Treatment or Trial

Individual participants may discontinue study treatment due to AEs, withdrawal of consent, or clinical judgment. The trial may be prematurely terminated for safety, feasibility, or funding reasons. All relevant ethics committees and regulatory bodies will be notified within 15 days of early termination.

#### Intervention Fidelity Monitoring Plan

To ensure both scientific rigor and pragmatic feasibility, we will implement a 3-part fidelity monitoring plan for this clinical trial.

##### Preintervention Training and Development

Research staff will undergo formal training in institutional research practices, including thorough review of the study protocol, which outlines the study approach, underlying evidence for the intervention, and research methods. Staff will attend dedicated training sessions to ensure a consistent understanding of their roles and how these fit within the broader intervention framework. Additionally, research staff will familiarize themselves with operations in relevant clinical areas such as the emergency department, wards, and ICU, where patients involved in the intervention will be managed.

##### Monitoring of Protocol Delivery

A PhD-trained coinvestigator will oversee fidelity monitoring focused on the core elements defined in the research protocol. This will involve using a standardized checklist to assess research staff interactions with patients, compliance with trial medication administration as per the protocol (ensuring adequate blinding), and auditing records of telephone calls made to patients for questionnaire completion.

##### Maintenance of Protocol Fidelity Over Time

Ongoing adherence to the core principles of the pragmatic trial intervention protocol will be maintained by the research nurse. A coinvestigator will audit patient interactions and randomly review 10 telephone call recordings every 3 months. The goal is to maintain at least 80% adherence to the protocol throughout the trial duration.

#### Consumer Engagement

This clinical trial involves a robust partnership between the public and researchers. Consumers were involved in advising on the research project, including its design and implementation. Recruitment efforts included invitations to former patients with CAP. Using the James Lind Alliance framework [25], we surveyed patients with CAP at Flinders Medical Centre to prioritize research questions. Early recovery from pneumonia was identified as the patients’ top health priority, followed by quick discharge from hospital, shaping our trial’s focus.

Consumer feedback also influenced trial logistics. A focus group meeting addressed concerns about additional blood samples, leading to the decision to overlap trial-specific investigations with routine blood collection. Strategies to improve recruitment and retention will be developed with patient input, ensuring the trial’s acceptability and feasibility. These will include simplifying participant information and consent form, increasing awareness through social media, and offering telehealth follow-ups.

For dissemination, we will develop plain-language summaries of the trial results with consumer input, ensuring that they are clear and actionable. Patient-centered knowledge translation plans will include educational materials to integrate successful interventions into clinical practice. This comprehensive consumer involvement will enhance the trial’s relevance, quality, and impact on patient care.

### Ethical Considerations

This study involves human participants and has received ethics approval from the Southern Adelaide Local Health Network Human Research Ethics Committee (approval 2025/HRE00407; approved on November 23, 2025). The study will be conducted in accordance with the principles of the Declaration of Helsinki and relevant Australian national guidelines for ethical conduct in human subject research.

#### Informed Consent

Written informed consent will be obtained from all participants before enrollment. For patients who are temporarily unable to provide consent due to illness, consent will be obtained from a legally authorized representative in accordance with guidance from the ethics committee.

#### Privacy and Confidentiality

Participant confidentiality will be strictly maintained throughout the study. All collected data will be recorded in secure electronic case report forms and stored on password-protected institutional servers. Each participant will be assigned a unique study identification number, and identifiable information will be stored separately from research data. Data used for analysis will be deidentified to protect participant privacy.

#### Participant Compensation

Participants will not receive financial compensation for taking part in this study. All study-related procedures will be conducted alongside routine clinical care where possible to minimize participant burden.

## Results

The VitCAP trial was registered with the Australian New Zealand Clinical Trials Registry under registration number ACTRN126250013614930.

Funding for the trial was awarded through the Southern Adelaide Local Health Network 2025 Enquiry Grant Round and 2025 Flinders Foundation Health Seed Grant. Following ethics approval and funding confirmation, trial preparation activities were undertaken, including finalization of the study protocol, development of electronic case report forms, preparation of study medication and placebo through the hospital pharmacy service, and training of research staff in study procedures and intervention delivery.

Participant recruitment is scheduled to commence in 2026 at a tertiary hospital in South Australia. Recruitment is planned over an estimated 18- to 24-month period, with a target sample size of 124 participants. This sample size was determined to provide 80% statistical power to detect a clinically meaningful difference in the primary outcome of time to clinical stabilization.

Follow-up of enrolled participants will take place at 30 days and 6 months after randomization. Completion of participant recruitment and follow-up is anticipated by 2028. Data cleaning and statistical analyses will commence after completion of follow-up, with the primary trial results expected to be disseminated through peer-reviewed publications thereafter in 2028.

## Discussion

### Anticipated Findings

The VitCAP trial is designed to evaluate whether sustained high-dose oral vitamin C supplementation improves clinical recovery and patient-centered outcomes in adults hospitalized with CAP. We hypothesize that vitamin C administration during the acute phase of illness and early recovery will shorten time to clinical stabilization and improve longer-term outcomes such as symptom burden, HRQOL, and health care use.

CAP remains a major cause of hospitalization, morbidity, and mortality worldwide despite advances in antimicrobial therapy and supportive care. Therefore, adjunctive treatments that are inexpensive, widely available, and biologically plausible are of considerable interest. Vitamin C plays a central role in immune function, endothelial integrity, and redox homeostasis, all of which are disrupted during acute pulmonary infection. Observational studies consistently demonstrate high rates of hypovitaminosis C among hospitalized patients with CAP, with deficiency associated with disease severity and adverse outcomes.

### Comparison With Prior Work

Previous interventional studies [14,16,17] evaluating vitamin C in pneumonia and sepsis have yielded heterogeneous results, largely due to differences in patient populations, dosing regimens, formulations, and abrupt therapy cessation. Most trials have focused on patients with sepsis who are critically ill, often using intravenous ascorbic acid and short treatment durations, and have not prioritized patient-centered outcomes such as symptom burden, recovery trajectory, or HRQOL. The VitCAP trial seeks to address these gaps by evaluating oral sodium ascorbate administered over an extended period in a broader population of hospitalized patients with CAP.

The primary outcome of time to clinical stabilization is clinically meaningful, reproducible, and closely linked to patient recovery and health care use. Secondary outcomes capture early response, longer-term symptoms, readmissions, and quality of life, thereby providing a comprehensive assessment of both short- and medium-term benefits. The inclusion of inflammatory biomarkers will also allow for the exploration of potential mechanistic pathways and identification of patient subgroups most likely to benefit from supplementation.

### Strengths and Limitations

This study has several strengths. The randomized, double-blind, placebo-controlled design minimizes bias, whereas the pragmatic oral supplementation strategy reflects an intervention that could be readily implemented in routine clinical practice. The extended treatment period allows for the evaluation of vitamin C supplementation not only during the acute phase of infection but also during early recovery following hospital discharge.

Several limitations should be acknowledged. First, the trial is powered to detect differences in time to clinical stabilization and may be underpowered to detect differences in mortality outcomes. Second, oral supplementation may limit peak plasma concentrations compared with intravenous administration, although it remains a pragmatic, widely applicable strategy. Third, although the trial includes biomarker analyses, it is not primarily designed to establish causal mechanistic pathways. Finally, the study is conducted within a single national health care system, which may affect generalizability.

### Future Directions and Implications

If effective, vitamin C supplementation could represent a low-cost, scalable adjunct to standard CAP management with potential applicability across diverse health care settings. The findings may also help inform future multicenter trials designed to evaluate vitamin C supplementation in broader patient populations and clarify the role of antioxidant therapies targeting host response pathways in pneumonia.

### Dissemination Plan

The findings of the VitCAP trial will be disseminated through publication in peer-reviewed journals and presentation at national and international scientific conferences. The results will also be communicated to clinicians and health care stakeholders to inform future clinical research and guideline development.

### Conclusions

The VitCAP trial is a rigorously designed, double-blind RCT evaluating high-dose oral vitamin C as an adjunctive therapy in hospitalized patients with CAP. By focusing on clinically meaningful outcomes, extended treatment duration, and mechanistic biomarkers, this study aims to address critical evidence gaps in the management of CAP. The results will provide important data to inform future clinical practice and guideline development.

## Appendix

Multimedia Appendix 1 Peer-review report by the Southern Adelaide Local Health Network (SALHN) Enquiry Grant, Government of South Australia.

Multimedia Appendix 2 SPIRIT checklist.

## Abbreviations

AE: adverse event
CAP: community-acquired pneumonia
CITRIS-ALI: Vitamin C Infusion for Treatment in Sepsis Induced Acute Lung Injury
CURB-65: confusion, urea, respiratory rate, blood pressure, and age ≥65 years
HRQOL: health-related quality of life
ICU: intensive care unit
LOS: length of stay
LOVIT: Lessening Organ Dysfunction With Vitamin C
RCT: randomized controlled trial
RR: risk ratio
SAE: serious adverse event
SPIRIT: Standard Protocol Items: Recommendations for Interventional Trials
VitCAP: Vitamin C in Community-Acquired Pneumonia

## Data Availability

No datasets were generated or analyzed during this study. Data generated from the VitCAP trial will not be publicly available due to ethical and privacy restrictions but may be made available from the corresponding author on reasonable request, subject to approval from the Southern Adelaide Local Health Network Human Research Ethics Committee.
