# Supplementary material for: Vitamin C Supplementation in Hospitalized Patients With Community-Acquired Pneumonia: Protocol for a Randomized Controlled Trial
Source: JMIR Res Protoc. 2026 Apr 29;15:e91037. doi: 10.2196/91037 (PMC13173059; doi:10.2196/91037)
Supplement: Multimedia Appendix 1 [file resprot_v15i1e91037_app1.pdf]

# 2024 SALHN Enquiry Grant Round – Applicant Feedback Sheet

**Title: The Impact of Vitamin C Supplementation in Hospitalised Patients with Community-Acquired Pneumonia: A Randomized Controlled Trial**

**Project Lead: Yogesh Sharma**

**Application ID Number: 2024EG04**

## Reviewer Feedback:

Reviewer 1

Well-written, but timeline and budget more than a touch ambitious on recruitment, and 10% attrition is likewise optimistic. Expect 3 patients/week -to reach 182 participants recruited means at least 60 weeks to just recruit, let alone complete the study. Means budget is probably underdone as coord role will be needed for longer. Affects impact score too. Consumer "pull" for project is a little unclear.

Reviewer 2

The application is well-written with very clear aims. However, there are some concerns as detailed in the following:

1) older multimorbid patients were mentioned as at a high risk of mortality. However, no strategies have been developed to address age and multimorbidity as confounding variables in the randomisation of participants.
2) The researchers described “stratified by clinical site” but did not explain the clinical site.

Reviewer 3

Great idea, will be low cost with big impact if hypothesis is right Given the potential adverse events associated with high dose vitamin C, should GI co-morbidity be an exclusion criterion? Daily monitoring of electrolytes, is daily bloods and monitoring standard of care in this patient cohort? Page 11 refers to infusion - treatment is oral? Daily recommended dose is 2grams, what is rational behind 3grams?
